# Job satisfaction, personality traits, and its impact on motivation among mental health workers

**DOI:** 10.4102/sajpsychiatry.v28i0.1801

**Published:** 2022-03-18

**Authors:** Oladipo A. Sowunmi

**Affiliations:** 1 Department of Clinical Services, Neuropsychiatric Hospital Aro, Abeokuta, Nigeria

**Keywords:** job satisfaction, personality, motivation, Aro, Abeokuta, mental health

## Abstract

**Background:**

Working in a resource setting that caters to people’s poor mental health is associated with increased vulnerability to physical, psychological, and social stressors that make motivation to work a difficult goal to attain. One way of viewing physical and social stressors in the workplace is to evaluate job satisfaction which has both intrinsic and extrinsic components. The personality of workers is a component of psychological wellbeing and this determines the way events and situations are perceived. Thus, the achievement of the mission and vision of an organisation will be dependent on the level of motivation of the employees which will be influenced by their predominant personality traits and the level of satisfaction at work.

**Aim:**

My study aimed to sought to highlight the relationship between motivation, job satisfaction and personality dimensions.

**Setting:**

The Neuropsychiatric Hospital, Aro, Abeokuta, Ogun State, Nigeria.

**Methods:**

Our study involved a cross-sectional study of staff showing the relationship between motivation, job satisfaction and personality traits among mental health workers. A total of 146 participants using systematic proportional sampling were analysed with a response rate of 67.3%. A Socio-demographic Questionnaire, Minnesota Satisfaction Questionnaire (Short Version), Big Five Inventory and the Multidimensional Work-Motivation Scale were administered to the participants. In the analysis, linear correlation and linear regression were used to determine the relationship between continuous variables (Normality was determined using kurtosis and skewness) while *t*-test was used to determine the relationship between categorical independent variables and continuous dependent variables.

**Results:**

The level of significance was set at < 0.05 while higher scores using the Multidimensional Work-Motivation Scale represented motivated participants and vis-a-vis. The socio-demographic variable was explored using descriptive statistics; the relationship between personality, job satisfaction and motivation were explored using *t*-test. Most of the participants were married (80.8%), female (60.3%), with at least tertiary education (63%) and with an occupational status of class I (76%). The mean age of the participants was 40.29 ± 8.27 with a mean length of service of 13.63 ± 8.49. The most dominant personality traits were agreeableness (97.3%) and conscientiousness (97.3%), and the least was neuroticism (55.5). High agreeableness (0.01), high conscientiousness (0.03), and high openness (0.01) were significant and positively correlated with motivation. The relationship between motivation and gender (*t* = 4.26; *p* ≤ 0.001) and occupational status were statistically significant (*t* = -3.59; *p* ≤ 0.001).

**Conclusion:**

To proffer a solution to poor motivation in the workplace, human resource department should give more focus to individuals with high scores in agreeableness, conscientiousness, and openness. This is because it appears that they are more likely to be motivated at work and likely to move the organisation to a greater height. Besides, those with high neurotic scores who have already been employed will require some form of psychological remodelling (therapy), so they can contribute meaningfully to the institution.

## Introduction

For any organisation to achieve its mission and vision, the employees must be motivated. However, the motivation of employees is influenced by their personality traits and their subjective and objective sense of job satisfaction.

Pencheon defined motivation as the extent to which tenacious effort is directed towards an organisational goal.^1^ Moon and his co-researchers also defined motivation as a set of energetic forces that have both intrinsic and extrinsic origins that go beyond an individual’s perspective of how work affects the way they behave and the biological, social and psychological component of these behaviours.^2^ In more succinct terms, motivation is the initiation of a specific behaviour with the aim to meet a perceived need. The fulfilment of organisational and personal goals results in an intrinsic or extrinsic reward. Intrinsic fulfilment is derived from within the individual, whereas extrinsic fulfilment pertains to rewards given by the organisational structure of a work institution.^3^

Studies of human motivation have postulated different opinions on how people become motivated. These include Maslow’s Hierarchy of Needs, Alderfer’s Theory, Herzberg’s Two-factor Theory and McClelland’s Acquired Needs Theory. This does not leave out the opinions of Skinner’s Reinforcement that focuses on external factors and those of Adam’s Equity Theory, Vroom’s Expectancy Theory, and Locke’s Goal-setting Theory which concentrates on internal factors. These theories postulate that to satisfy this intrinsic and extrinsic fulfilment (motivation) within a work environment, the workforce must have a sense of satisfaction with the role they play in their institution (job satisfaction), and their perception of the workplace will be influenced by their personality.^2,3,4^

Furthermore, job satisfaction can be defined as a prolonged, pleasurable and positive state of feeling based on an individual’s job or workplace experiences.^3^ The level of productivity in any institution has a direct correlation with employees’ feeling of stress and job satisfaction. Thus, staff with low stress and high workplace satisfaction have greater competitive edge while those with high stress and low satisfaction have poor competitive edge. As a result, organisations must pay close attention, not only to the physical and social dispositions of workers but also to the psychological aspects of employees which include their personality.^4^

The evaluation of an employee’s duties and possible benefit is coloured by their personality. What is important is that people’s personality is not only quantitative but qualitative. Hence, while the terms of reference of a job may appear tedious to one individual, it may be simple to another, not because the volume of activities are reduced but because their personality has input on how things are perceived. To this end, whether or not a challenging work gets appropriate value system such as opportunities to progress in one’s chosen career or having a desired and competent supervisor or having cooperative co-workers, the determinant of satisfaction is dependent on an individual’s personality trait.^3^

Theories on personality traits opine that different people have several dispositions to how they respond to physical, psychological and social environments. The understanding of this opinion allows managers to have a better understanding on how to communicate with staff in the workplace and how best to approach job sizing. Moreover, personality traits may serve as key indicators of other parts of an individual’s personal life of which motivation and job satisfaction are paramount.^1,2,3,5^

In any healthcare setting and especially in the mental health sector, the main objectives are to ensure that mental health services are not only available in tertiary and secondary care but are also available in primary healthcare settings. Also, these services should be affordable, accessible and as modern as possible. In order to achieve these objectives, mental and general health workforce are needed in large numbers so that they can function in all the needed multidisciplinary cadre and in all the vital geographical regions. One of the things that inhibit how these objectives are met is the lack of explicit human resource management policies, especially in most less developed countries including Nigeria. Policies should focus on mental health professions such that physical, psychological and social needs are met and also noting that these workforce are heterogeneous with differing economic needs. Thus, health organisations are faced with external pressures that cannot be effectively met without appropriate adjustments. As a result, motivation, job satisfaction and personality traits remain crucial to developing health policies in low to middle-income countries like Nigeria.

The lack of commitment in the healthcare sector, especially mental health services in less developed countries like Nigeria is as a result of the stigma associated with mental illness and mental health workers. To overcome the overwhelming stigma associated with this industry, the workforce must be motivated if the minimum standard of service will be made available.^3^

Despite this fact, few studies have looked into the topic of motivation among employees in the mental health industry in Nigeria. Moreover, there is little or no literature on the relationship between motivation, job satisfaction and employees’ personality traits. The dwindling economic status or power of most mental health workers has made it difficult to improve satisfaction at the workplace with possible harmful gene-environment interaction with healthcare workers’ personalities especially in low- and middle-income societies like Nigeria.^1^

In addition, there is a general consensus that there may be risk factors underlying the relationship between personality traits and job satisfaction, although these have been scarcely studied.^1,2,3,4,5^ To address this, earlier writers have called for a synergy of all available theories evaluating personality traits in relation to motivation and job satisfaction. In line with this recommendation, this study posits that there is a direct correlation between motivation, personality traits and job satisfaction. That is, individuals with certain personality traits are more likely to be motivated and may perceive their lives as meeting the gold standard of the community, family living, personal health and career.^1,2,3,4,5^ This is because motivated individuals enjoy exploration that leads to doing something new and seeking new challenges, with the hope of proffering meaningful substance to life. Investigating motivation in relation to personality traits and workplace satisfaction gives us opportunity to know ‘what’ personality traits are associated with job satisfaction and ‘why’ individuals with these personality traits perceive their job as more satisfying. By so doing, my study will stimulate research beyond just the Big Five personality traits to explore motivation, personality traits and job satisfaction as complex and interrelated factors needed for the efficient and effective running of a mental health institution.^1,2,3,4,5^

## Materials and methods

This involved a cross-sectional study showing the relationship between motivation, job satisfaction and personality traits among mental health workers. My study was carried out at the Neuropsychiatric Hospital, Aro, Abeokuta, Ogun State, between September and October 2020. The hospital initially started at its annexe in 1944 as an asylum for soldiers who were repatriated home due to mental illness after the Second World War. The current main site, Aro, was built in 1954 when the need for a modern psychiatric hospital arose. The hospital renders services to patients from all over Nigeria and from neighbouring West African countries. It has a total capacity of 546 beds for inpatient care, 153 beds at the main hospital and 393 beds at the Lantoro annexe. As of March 2020, the total staff strength of the Neuropsychiatric Hospital, Aro, Abeokuta was 500.

### Sample size and sampling technique

The sample size formula used in epidemiological studies for calculating the mean of a sample from a large population described by Cochran^6^ was used in my study. A total of 217 electronic questionnaires were administered/sent; however, only 146 responses could be analysed given a response rate of 67.3%. Using a proportionate probability representation, the number of staff recruited from each unit/department in the hospital was calculated based on the fraction they contribute to the total sample population as highlighted below.

### Data collection

Based on the fraction that each unit contribute to the entire data population, the systematic sampling frequency was calculated by dividing the unit population by the number they contributed to the sample population. The first participant was picked through random sampling method before systematic random sampling was applied. Subsequently, a Socio-Demographic Questionnaire, Minnesota Satisfaction Questionnaire (Short Version) and the Big Five Personality Inventory were administered.^7,8^

### Data analysis

Data were analysed using the Statistical Package for Social Science (SPSS version 25), Computer Software. Data was presented in frequency tables for descriptive statistics. The level of significance was set at *p* < 0.05. Some socio-demographic variables and clinical variables were regrouped to make them categorical variables and to eliminate empty cells during analysis. The patient’s occupation was classified according to the system of Boroffka and Olatawura as follows^9^. Group I consists of professionals with university degrees (doctors, lawyers, teachers, scientists, and high government officers). Group II consists of professionals without university degrees (teachers, administrators, high clerical and supervisory personnel, large-scale farmers, entrepreneurs, and armed forces officers). Group III consists of clerks, motor vehicle drivers, mechanics, tailors, butchers, soldiers, policemen and small-scale entrepreneurs. Group IV consists of cooks, barbers, domestic servants, gas station attendants, goldsmiths, palm-wine tappers, and small-scale farmers. Group V includes labourers and petty traders. Group VI includes full-time housewives, unemployed educated youths, and apprentices. For ease of analysis, the groups were reclassified into Group 1 and Group 2–6. Also, the level of education was reclassified into primary/secondary/diploma and tertiary/postgraduate education. Linear correlation and linear regression were used to determine the relationship between continuous variables (Normality was determined using kurtosis and skewness), while *t*-test was used to determine the relationship between categorical independent variables and continuous dependent variables.

### Ethical considerations

Approval to conduct the study was obtained from the Neuropsychiatric Hospital, Aro Research Ethics Committee, reference number: PR0/14/17.

## Result

### Response rate

A total of 217 questionnaires were distributed, but only 146 were returned giving a response rate of 67.3%.

### Socio-demographic characteristics of study participants

The age range of the respondents was 18–58 years with a mean (s.d.) of 40.29 (8.27) years. Other details can be found in Table 1 and Table 2.

**TABLE 1:** Socio-demographic (categorical variables) characteristics of participants.

Variables	*n*	%
**Gender**		
Male	58	39.7
Female	88	60.3
**Marital status**		
Without a partner	28	19.2
Married	118	80.2
**Level of education**		
Primary/Secondary/Diploma	54	37.0
Tertiary/Postgraduate	92	63.0
**Employment status**		
Permanent/Full-Time	130	89.0
Contract	16	11.0
**Occupational status**		
Group 1	111	76.0
Group 2–6	35	24.0

**TABLE 2:** Socio-demographic (continuous variables) characteristics of participants.

Central tendency	Continuous variables								
	Age	LOS	MSQT	ESS	ASS	CSS	NSS	OSS	MWMST
Mean	40.29	13.63	48.05	23.84	34.08	33.64	17.82	35.05	71.52
s.d.	8.27	8.49	12.05	4.17	6.42	6.63	4.77	5.78	19.62
Skewness	−0.33	1.002	0.465	0.244	−0.474	−0.275	−0.050	−0.44	0.004
SES	0.20	0.201	0.201	0.201	0.201	0.201	0.201	0.201	0.201
Kurtosis	0.147	0.642	0.256	0.202	0.387	0.037	−0.048	0.661	0.210
SEK	0.399	0.399	0.399	0.399	0.399	0.399	0.399	0.399	0.399
Minimum	18	1	21	15	9	9	7	17	19
Maximum	58	35	84	37	45	45	32	50	121

s.d., standard deviation; SES, standard error of skewness; SEK, standard error of kurtosis; LOS, length of service; MSQT, Minnesota Satisfaction Questionnaire total score; ESS, extraversion subscale score; ASS, agreeableness subscale score; CSS, conscientiousness subscale score; NSS, neuroticism subscale score; OSS, openness subscale score; MWMST, multidimensional work motivation scale total score.

### Personality dimensions among study participants

Table 3 shows the personality dimensions of study participants.

**TABLE 3:** Personality dimensions among study participants.

Variables	*n*	%
**Extraversion subscale score**		
Low	32	21.9
High	114	78.1
**Agreeableness subscale score**		
Low	04	2.7
High	142	97.3
**Conscientiousness subscale score**		
Low	04	2.7
High	142	97.3
**Neuroticism subscale score**		
Low	65	44.5
High	81	55.5
**Openness subscale score**		
Low	08	5.5
High	138	94.5

#### Relationship between motivation and sociodemographic variables, job satisfaction and personality

Table 4 shows the relationship between motivation and job satisfaction, age, and personality dimensions.

**TABLE 4:** Relationship between motivation and job satisfaction, age, and personality dimension.

Correlations	Correlation	MSQ T	MWMST	Age	LOS	ESS	ASS	CSS	NSS	OSS
MWMST	PC	−0.16	1	0.15	−0.06	−0.034	0.207	0.19	−0.002	0.21
	*P*	0.05	-	0.08	0.49	0.69	0.01	0.03	0.98	0.01

PC, Pearson correlation; *P, p*-value; LOS, length of service; MSQT, Minnesota Satisfaction Questionnaire total score; ESS, extraversion subscale score; ASS, agreeableness subscale score; CSS, conscientiousness subscale score; NSS, neuroticism subscale score; OSS, openness subscale score; MWMST, multidimensional work motivation scale total score.

#### Relationship between motivation and categorical variables

Table 5 shows the relationship between motivation and gender, marital status, level of education, employment status and occupational status.

**TABLE 5:** The relationship between motivation and gender, marital status, level of education, employment status and occupational status.

Variables		LTEV					TEM		95%CI	
		*F*	*p*	*t*	df	*p*	MD	SED	Lower	Upper
Independent samples test (marital status)										
MWMST	EVA	2.77	0.09	−0.73	144	0.46	−3.03	4.13	−11.19	5.13
	EVNA			−0.83	48.03	0.41	−3.03	3.66	−10.38	4.32
Independent samples test (level of education)										
MWMST	EVA	0.89	0.35	0.97	144	0.33	3.26	3.36	−3.39	9.91
	EVNA			0.94	101.42	0.35	3.26	3.46	−3.61	10.13
Independent samples test (employment status)										
MWMST	EVA	0.17	0.68	−0.37	144	0.71	−1.94	5.21	−12.25	8.36
	EVNA			−0.37	18.79	0.72	−1.94	5.26	−12.96	9.07
Independent samples test (occupational status)										
MWMST	EVA	0.08	0.78	−3.59	144	**<0.001**	−13.15	3.66	−20.37	−5.92
	EVNA			−3.57	56.47	0.001	−13.15	3.68	−20.52	−5.78
Independent samples test (gender)										
MWMST	EVA	1.52	0.22	4.26	144	<0.001	13.35	3.14	7.15	19.56
	EVNA			4.19	115.33	0.000	13.35	3.19	7.04	19.67

Bold indicates significant *P*-value.

* *p, p*-value; LOS, length of service; MSQT, Minnesota Satisfaction Questionnaire total score; ESS, extraversion subscale score; ASS, agreeableness subscale score; CSS, conscientiousness subscale score; NSS, neuroticism subscale score; OSS, openness subscale score; MWMST, multidimensional work motivation scale total score; LTEV, Levene’s test for equality of variances; TEM, t-test for equality of means; MD, mean difference; SED, standard error difference; 95% CI, 95% confidence interval; EVA, equal variances assumed; EVNA, equal variances not assumed.

## Discussion

The response rate observed in my study was lower than that of two previous studies conducted among mental health workers in North Central (90%)^10^ and South West (71.5%)^11^ zones of Nigeria. However, the response rate in my study was close to the study conducted in the southwest zone. The study in the southwest zone was conducted in the same state (Ota, Ogun State)^11^ as the current study. A possible explanation is that my current study was carried out electronically unlike the two previous studies where paper questionnaires were administered. The use of electronic questionnaires is new to the study participants and may have increased the attrition rates.

This study reveals a high mean score for motivation (71.52), which is similar to a study^12^ conducted among a cross-section of the federal health institutions in the southern states of Nigeria. Furthermore, the finding of the high mean score for motivation among health workers was also similar to a study among health workers in the United Kingdom.^13^ This finding is opposite to the anecdotal reports that suggest that the majority of federal government workers are dissatisfied with their work. The difference is best understood if the concept of motivation is used to throw more light on the different section of the federal health institution in the southern states of Nigeria.

Motivation is believed to have two core dimensions, namely, intrinsic motivation (which has to do with the fact that activities are done because they are enjoyed) and extrinsic motivation (which associates one’s willingness to be involved in an activity based on his or her values and beliefs). Furthermore, motivation could be viewed in the following dimensions: Amotivation, where workers have no intention to engage in work; External regulation, where workers do an activity only to obtain a reward; Introjected regulation, where workers are motivated by self-worth and related factors; Identified regulation, where workers recognise the value of the activity and perceive it as their own; Integrated regulation, where the value of the activity is part of a worker’s identity; Intrinsic motivation, where workers find the task inherently enjoyable, challenging, and/or a form of self-actualisation. In the light of this information workers in a mental health facility may enjoy their work and thus have an innate interest in their work or their motivation thrust from external, introjected, identified, and integrated regulations. This may explain the finding of high motivation scores despite the economic plight of the nation.

The mean score for job satisfaction was 48.05; this was lower than what was reported within the same facility where my present study was conducted.^14^ However, it was similar to other reports in the United Kingdom. The difference may be related to the fact that my study reviewed both clinical and non-clinical staff of the hospital. It appears that non-clinical staff may be less satisfied with their job when compared with the clinical staff of the hospital. The concept of job satisfaction can be viewed as intrinsic and extrinsic both of which ultimately determines whether a person is satisfied with their job. It is possible that intrinsic factors may be more contributory with regard to clinical staff when compared with non-clinical staff, hence, the reason for the difference. Future studies should involve both clinical and non-clinical staff of a mental health facility to establish a trend.

The author’s study showed that the most prevalent personality dimensions were agreeableness and conscientiousness. Agreeableness is related to how well people get along with others. The trait of agreeableness includes people who are warm-natured, trustworthy, cooperative, compliant, compassionate, and supportive. They are also altruistic, modest, patient, moderate, tactful, polite, sensitive, amiable, cheerful and considerate.^15^ People who have high agreeableness tend to be well-liked, respected, and sensitive to the needs of others. They likely have few enemies, are sympathetic, and affectionate to their friends and loved ones, as well as sympathetic to the plights of strangers. People on the low end of the agreeableness spectrum are less likely to be trusted and liked by others. They tend to be callous, blunt, rude, ill-tempered, antagonistic, and sarcastic.

On the other hand, conscientiousness encompasses dependability, responsibility, ambition, thoroughness, self-discipline, predictableness, resourcefulness, diligence, dynamism, planning, achievement orientation and deliberation. It entails following established rules and includes people who are persistent and organised. A person who is low in conscientiousness is much more likely to procrastinate, to be flighty, impetuous, and impulsive. Considering this information, the staff of this institution are systematic, purposeful individuals who are soft-hearted and forgiving, determined, strong-willed and helpful. This finding was in contrast to a finding in Istanbul, among nurses and physicians^16^ where neuroticism and extraversion were the most prevalent personality dimensions. They opined that agreeableness and conscientiousness were better traits to promote cooperation in a health facility with job satisfaction. Conflicts were suggested to be less in a workplace where agreeableness and conscientiousness predominated.

The author found a significant relationship between motivation and agreeableness, conscientiousness, and openness. This is interpreted to mean that the staff of the Neuropsychiatric Hospital, Aro, Abeokuta, could be viewed as individuals with communications skills who build connections between different parties and are willing to learn from every opportunity. Besides, they are goal-directed and think outside the box and as managers, they have good learning and performance orientation.^17^

The results showed a significant relationship between female gender and motivation and thus buttressed the opinion that female gender is a significant factor in workplace motivation.^18^ The female gender is believed to be more concerned about social security at the workplace; they are more concerned about what and who talks about them and are willing to compromise such that cordial relationships at the workplace take pre-eminence over their take-home pay. Women are believed to be home builders and they tend to promote home features within the workplace. These features have been suggested not to be the main concern of the male gender as they are believed to be more self-confident and concerned about their salaries. Furthermore, my study observed that occupational status was a significant predictor of motivation. In the author’s study, occupational status is a function of the educational status and economic earning power of the staff. Thus, those with tertiary education were more motivated compared to those with lesser educational experience. This is believed to be related to the fact that motivation comes with the opportunity to improve and be successful. This is a probable explanation of the findings of my study.

In conclusion, the author observed that high motivation among mental health workers at the Neuropsychiatric hospital, Aro, Abeokuta was significantly related to the following socio-demographic variables: gender and occupational status. Also, high motivation in this mental health facility was directly related (positive correlation) to the following personality traits: agreeableness, conscientiousness, and neuroticism.

In the light of the foregoing, the author recommends that, firstly, motivation in the workplace is important and it is heightened by factors such as being female, having tertiary education, class I occupational status and being married. It will be important for government and policymakers to focus their attention on the male gender, those who are not married, staff with an educational status lower than tertiary education and people with an economic class lower than one. Secondly, to proffer a solution to the poor motivation in the workplace, the human resource department should focus more on individuals with high scores in agreeableness, conscientiousness, and openness. It appears that they are more likely to be motivated at work and likely to move the institution to a greater height. Besides, those with high neurotic scores who have already been employed will require some form of psychological remodelling to allow them contribute meaningfully to the institution.

Finally, every workplace should provide mental health services for its employees with a view of promoting mental health and ensuring psychological stability within the workplace. It is important to note that my study is not without its limitations. The limitations of my study include the following: my study is a cross-sectional study and therefore, it was not possible to establish the causality in the relationship between motivation and personality dimensions and job satisfaction. The use of self-reported questionnaires to assess motivation, personality dimension and job satisfaction may be associated with recall bias and it is not certain how this could have affected the findings. Therefore, future studies should consider a longitudinal study as a more realistic and reliable approach to determining causality. Furthermore, qualitative studies may be used to overcome recall bias which will provide more information on the constructs of motivation, personality dimension and job satisfaction.
